# Factors impact on insufficient nutrition and effects of timely adequate nutrition support on patient outcomes in adult intensive care patients

**DOI:** 10.1186/2197-425X-3-S1-A585

**Published:** 2015-10-01

**Authors:** Y Savran, M Limon, ME Tokur, B Comert

**Affiliations:** Dokuz Eylul University Faculty of Medicine, Internal Medicine Intensive Care Unit, Izmir, Turkey; Dokuz Eylul University Faculty of Medicine, Internal Medicine, Izmir, Turkey

## Introduction

Most of the studies comparing effects of enteral and parenteral nutrition on morbidity and mortality have been performed on surgery patient groups or in mixed intensive care unit patients. Numbers of studies performed in medical intensive care units are limited and they all include few number of patients.

## Objectives

In the present study we aimed to analyze association of adequacy of nutrition support with infectious complications, length of stay in intensive care unit and hospital, and mortality in medical intensive care unit patients and causes of insufficient nutrition in patients who are not met target daily caloric intake.

## Methods

Data of patients who were hospitalized in medical ICU between January 2012 and December 2013 were reviewed retrospectively. Patients older than 18 years of age and who had been dependent to mechanical ventilation for at least 3 days were included. 220 patients were determined after retrospective review of patient files with laboratory and imaging results.

## Results

133 male and 87 female patients were included.151 patients reached the target caloric intake.Mean target calorie was 1579 kcal/d in the adequate caloric intake group, and 1739 kcal/d in insufficient caloric intake group (p < 0.001).The number of comorbidities in both groups were similar (1.91 ± 1.07/1.87 ± 1.00; p >0.05).According to logistic regression analysis parenteral nutrition and lymphoma were found to be major independent risk factors for insufficient calorie intake. The most common cause for cessation of nutrition was detection of a residual. Nosocomial infection frequency in adequate and insufficient caloric intake groups were similar, respectively. Enteral nutrition was the most effective feeding way in adequate caloric intake group (p < 0.002). Parenteral nutrition and immunosuppressive treatment were major independent risk factors for mortality based on logistic regression analysis.Mortality of patients in adequate and insufficient caloric intake groups was 53% and 76%, respectively (p < 0.002).

## Conclusions

Enteral nutrition in critical care patients is safe and effective. Patients who were fed enterally were more associated with reaching the target caloric intake.It is far more difficult to reach target caloric intake by parenteral nutrition and it is associated with an increase in mortality.

More effort should be sought for the continuity of enteral nutrition.Adequate amounts of nutrition significantly decreases mortality.
